# Synthesis, Characterization and *In Vitro* Antitumour Activity of Di-n-Butyl, Tri-n-Butyl and Triphenyltin 3,6-Dioxaheptanoates and 3,6,9-Trioxadecanoates

**DOI:** 10.1155/MBD.1998.189

**Published:** 1998

**Authors:** Martine Kemmer, Marcel Gielen, Monique Biesemans, Dick de Vos, Rudolph Willem

**Affiliations:** 1 Université Libre de Bruxelles Service de Chimie Organique Av. F. D. Roosevelt, 50 Bruxelles B-1050 Belgium; 2 Free University of Brussels VUB Pleinlaan 2 AOSC Unit Faculty of Applied Sciences Brussel B-1050 Belgium; 3 Free University of Brussels VUB Pleinlaan 2 High Resolution NMR Center HNMR Brussel B-1050 Belgium; 4 Medical Department Pharmachemie B. V. Haarlem NL-2003 RN The Netherlands

## Abstract

A series of di- and triorganotin 3,6-dioxaheptanoates and 3,6,9-trioxadecanoates were synthesized and characterized by ^1^H, ^13^ and ^117^Sn NMR, electrospray mass and ^119m^Sn Mössbauer spectroscopy, as well as elemental analysis. Their *in vitro* antitumour activity against seven tumoural cell lines of human origin, two breast cancers (MCF-7, EVSA-T), a colon carcinoma (WiDr), an ovarian cancer (IGROV), a melanoma (M 19 MEL), a renal cancer (A 498) and a non small cell lung cancer (H 226), is reported. They are characterized by similar inhibition doses ID_50_ as the analogous di- and triorganotin derivatives of 4-carboxybenzo-15-crown-5 and -18-crown-6 and in some cases by much lower ID_50_ values than clinically used reference compounds such as doxorubicine and methotrexate.


### SYNTHESIS, CHARACTERIZATION AND *IN VITRO* ANTITUMOUR ACTIVITY OF DI-n-BUTYL, TRI-n-BUTYL AND TRIPHENYLTIN 3,6-DIOXAHEPTANOATES AND 3,6,9-TRIOXADECANOATES

Martine Kemmer[1], Marcel Gielen*[1,2a], Monique Biesemans[2a,b], Dick de Vos[3] and Rudolph Willem[2a,b]

[1] Université Libre de Bruxelles, Service de Chimie Organique, Av. F. D. Roosevelt, 50, B-1050 Bruxelles, Belgium
[2] Free University of Brussels VUB, Pleinlaan 2, B-1050 Brussel, Belgium
[a] AOSC Unit, Faculty of Applied Sciences
[b] High Resolution NMR Center HNMR
[3] Medical Department, Pharmachemie B. V., NL-2003 RN Haarlem, The Netherlands

### Abstract
A series of di- and triorganotin 3,6-dioxaheptanoates and 3,6,9-trioxadecanoates were synthesized and characterized by $^{1}H$, $^{13}C$ and $^{117}Sn$ NMR, electrospray mass and $^{119m}Sn$ Mössbauer spectroscopy, as well as elemental analysis. Their *in vitro* antitumour activity against seven tumoural cell lines of human origin, two breast cancers (MCF-7, EVSA-T), a colon carcinoma (WiDr), an ovarian cancer (IGROV), a melanoma (M19 MEL), a renal cancer (A 498) and a non small cell lung cancer (H 226), is reported. They are characterized by similar inhibition doses $ID_{50}$ as the analogous di- and triorganotin derivatives of 4-carboxybenzo-15-crown-5 and -18-crown-6 and in some cases by much lower $ID_{50}$ values than clinically used reference compounds such as doxorubicine and methotrexate.

### Introduction
Many di-n-butyl, tri-n-butyl and triphenyltin carboxylates display interesting antitumour activities *in vitro* against tumour cell lines of human origin [1,2]. As early as 1985, Atassi suggested that the usually low water solubility of organotin compounds might be the major drawback to the improvement of their antitumour properties [3]. One possibility to increase water solubility is to replace methylene groups in a polymethylenic chain by oxygen atoms [4]. Accordingly, some new organotin derivatives of 3,6-dioxaheptanoic and 3,6,9-trioxadecanoic acid were synthesized. This report presents their synthesis, characterization and *in vitro* antitumour activity.

### Results and discussion
*Synthesis*

For the triorganotin polyoxaalkanoates, the condensation is carried out in benzene using triphenyltin hydroxide respectively tri-n-butyltin acetate and either 3,6-dioxaheptanoic or 3,6,9-trioxadecanoic acid (equations 1 and 2) [2-5].

$$(C_6H_5)_3SnOH + RCOOH \longrightarrow (C_6H_5)_3SnOCOR + H_2O \quad (1)$$

$$Bu_3SnOCOCH_3 + RCOOH \longrightarrow Bu_3SnOCOR + CH_3COOH \quad (2)$$

For the diorganotin polyoxaalkanoates, di-n-butyltin oxide first reacts with n-propanol to form tetrabutyldipropoxydistannoxane (equation 3) [6].

$$2\ Bu_2SnO + 2\ PrOH \longrightarrow (PrOSnBu_2)_2O + H_2O \quad (3)$$

Subsequently, the polyoxaalkanoic acid is added at room temperature to the tetrabutyldipropoxydistannoxane in the desired molar ratio. A molar ratio 1/1 generates the corresponding bis[di-n-butyl(polyoxaalkanoato)tin]oxide (equation 4); a molar ratio 2/1 leads to the di-n-butyitin bis(polyoxaalkanoate) (equation 5).

$$2\ (PrOSnBu_2)_2O + 4\ RCOOH \longrightarrow \{[Bu_2(RCOO)Sn]_2O\}_2 + 4\ PrOH \quad (4)$$

$$(PrOSnBu_2)_2O + 4\ RCOOH \longrightarrow 2\ Bu_2Sn(OCOR)_2 + 2\ PrOH + H_2O \quad (5)$$

Sephadex LH-20 chromatography proved to be very efficient to separate the bis[di-n-butyl(polyoxaalkanoato)tin]oxides and tributyl- or triphenyltin polyoxaalkanoate from the starting material. The di-n-butyltin bis(polyoxaalkanoates) were hydrolyzed on Sephadex LH-20 into bis[di-n-butyl(polyoxaalkanoato)tin]oxides (equation 6). Hydrolysis was also observed in the presence of wet ethanol, diorganotin dicarboxylates often undergoing partial hydrolysis to form the dimeric distannoxanes [7].

$$4\ Bu_2Sn(OCOR)_2 + 2\ H_2O \longrightarrow \{[Bu_2(RCOO)Sn]_2O\}_2 + 4\ RCOOH \qquad (6)$$

The compounds synthesized are depicted in figure 1.

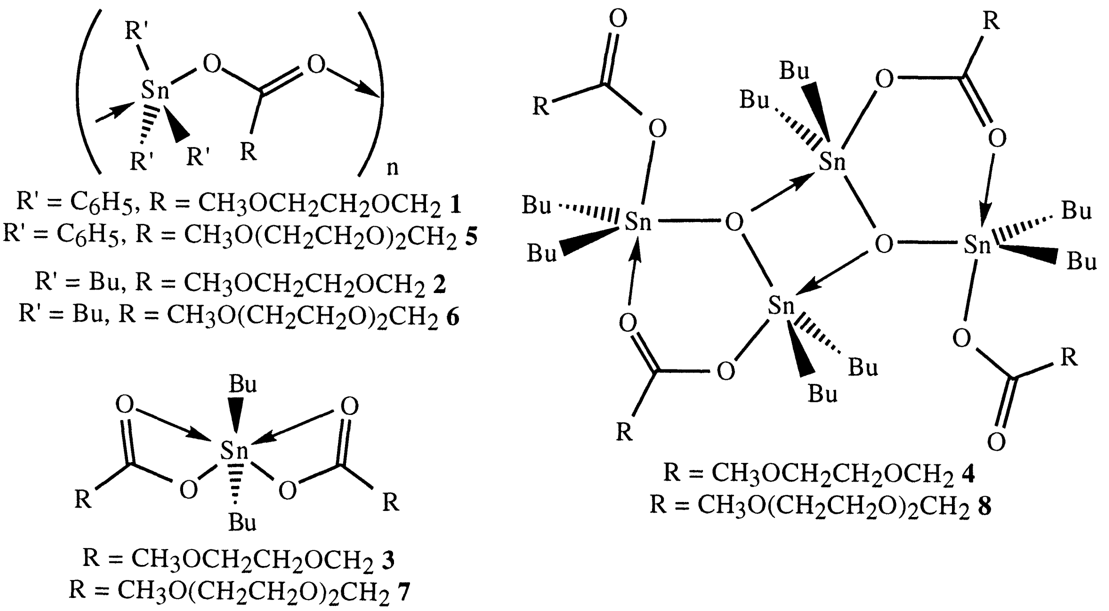

*figure 1*

| | **1** | | | **2** | | | **3** | | | **4** | | |
|---|---|---|---|---|---|---|---|---|---|---|---|---|
| CH(o) | 7.7-7.8 | m | [65] | - | | | - | | | - | | |
| CH(m)(p) | 7.4-7.5 | m | | - | | | - | | | - | | |
| $CH_2(2)$ | 4.25 | s | | 4.09 | s | | 4.16 | s | | 3.95 | s | |
| $CH_2(4)$ | 3.7-3.8 | m | | 3.6-3.7 | m | | 3.6-3.8 | m | | 3.6-3.7 | m | |
| $CH_2(5)$ | 3.5-3.6 | m | | 3.5-3.6 | m | | 3.5-3.6 | m | | 3.5-3.6 | m | |
| $CH_3(7)$ | 3.35 | s | | 3.36 | s | | 3.36 | s | | 3.34 | s | |
| HOH | - | | | ~2.2 | bs | | - | | | ~2.0 | s | |
| $CH_2(\beta)$ | - | | | 1.5-1.6 | m | | 1.6-1.7 | m | | 1.5-1.7 | m | |
| $CH_2(\alpha)$ | - | | | 1.2-1.4 | m | | 1.6-1.7 | m | [100] | 1.3-1.5 | m | |
| $CH_2(\gamma)$ | | | | 1.2-1.4 | m | | 1.34 | tq | (7, 7) | 1.30 | tq | (7, 7) |
| $CH_3(\delta)$ | - | | | 0.88 | t | (7) | 0.87 | t | (7) | 0.8-0.9 | m | |

Table 1: $^1H$ NMR data in $CDCl_3$ of compounds **1** to **4**; chemical shifts in ppm with respect to TMS; coupling constants in Hz, $^nJ(^1H-^1H)$ in parentheses, $^nJ(^1H-^{117/119}Sn)$ between square brackets. Abbreviations: s = singlet; m = complex pattern; b = broad; t = triplet; tq = triplet of quartets.

*Characterization*

NMR spectroscopy

All compounds were characterized by $^1H$, $^{13}C$, and $^{117}Sn$ NMR in $CDCl_3$. The $^1H$ NMR data are listed in tables 1 and 2. The proton chemical shifts of the carboxylate moiety were assigned by $^1H$-$^{13}C$ HMBC and HMQC experiments. The methylenic H(4), H(5) and H(7) proton resonances of compounds **1-5** appear as non overlapping patterns, those of compounds **6-8** do not. $^3J(^1H-^{117/119}Sn)$ coupling constants could be determined for compounds **1** and **5**, and $^2J(^1H-^{117/119}Sn)$ coupling constants, for compounds **3** and **7**.

The $^{13}C$ NMR data are given in tables 3 and 4. Signal assignment was carried out by $^1H$-$^{13}C$ HMQC and HMBC experiments. $^{13}C$ resonances of tri-n-butyltin and triphenyltin moieties were assigned straightforwardly from the $^{n}J(^{13}C-^{117/119}Sn)$ coupling constants [8,9]. Compounds **4** and **8** show pairs of $^{13}C$ resonances for each carbon type of the di-n-butyltin moieties. These are broad, precluding the observation of $^{n}J(^{13}C\ ^{117/119}Sn)$ satellites.

| | **5** | | | **6** | | | **7** | | | **8** | | |
|---|---|---|---|---|---|---|---|---|---|---|---|---|
| CH(o) | 7.7-7.8 | m | [60] | - | | | - | | | - | | |
| CH(m)(p) | 7.4-7.5 | m | | - | | | - | | | - | | |
| $CH_2(2)$ | 4.22 | s | | 4.09 | s | | 4.15 | s | | 3.96 | s | |
| $CH_2(4)$ | 3.7-3.8 | m | | 3.6-3.8 | m | | 3.6-3.8 | m | | 3.6-3.7 | m | |
| $CH_2(5)$ | 3.6-3.7 | m | | 3.6-3.8 | m | | 3.6-3.8 | m | | 3.6-3.7 | m | |
| $CH_2(7)$ | 3.5-3.6 | m | | 3.6-3.8 | m | | 3.6-3.8 | m | | 3.6-3.7 | m | |
| $CH_2(8)$ | 3.4-3.5 | m | | 3.5-3.6 | m | | 3.5-3.6 | m | | 3.5-3.6 | m | |
| $CH_3(10)$ | 3.34 | s | | 3.36 | s | | 3.35 | s | | 3.34 | s | |
| HOH | - | | | ~1.9 | b | | - | | | ~1.9 | b | |
| $CH_2(\beta)$ | - | | | 1.5-1.7 | m | | 0.6-0.8 | m | | 1.57 | tt | (7, 7) |
| $CH_2(\alpha)$ | - | | | 1.2-1.4 | m | | 0.6-0.8 | m | [102] | 1.4-1.5 | m | |
| $CH_2(\gamma)$ | - | | | 1.2-1.4 | m | | 1.35 | tq | (7, 7) | 1.27 | tq | (7, 7) |
| $CH_3(\delta)$ | - | | | 0.89 | t | (7) | 0.88 | t | (7) | 0.8-0.9 | m | |

Table 2: $^{1}H$ NMR data in $CDCl_3$ of compounds **5** to **8**, see legend table 1.

The $^{117}Sn$ NMR data are reported in table 5. Compounds **1**, **2**, **3**, **5**, **6** and **7** exhibit one single $^{117}Sn$ resonance. Compounds **4** and **8** exhibit two $^{117}Sn$ resonances of equal intensities, resulting from the dimeric structure characterized by endocyclic and exocyclic tin atoms (see figure 1).

| | **1** | | **2** | | **3** | | **4** |
|---|---|---|---|---|---|---|---|
| C(1) | 176.5 | | 175.2 | | 178.3 | | 174.9 |
| CH(i) | 137.7 | | - | | - | | - |
| CH(o) | 136.8 | [49] | - | | - | | - |
| CH(p) | 130.2 | [13] | - | | - | | - |
| CH(m) | 128.9 | [62/65] | - | | - | | - |
| $CH_2(5)$ | 72.0 | | 71.9 | | 71.8 | | 71.8 |
| $CH_2(4)$ | 70.6 | | 70.4 | | 70.7 | | 70.2 |
| $CH_2(2)$ | 69.0 | | 69.0 | | 68.6 | | 69.8 |
| $CH_3(7)$ | 59.0 | | 59.0 | | 59.0 | | 58.9 |
| $CH_2(\beta)$ | - | | 27.8 | [20] | 26.5 | [34] | 27.5 |
| | | | | | | | 27.2 |
| $CH_2(\gamma)$ | - | | 27.1 | [64/67] | 26.3 | [98/102] | 26.8 |
| | | | | | | | 26.7 |
| $CH_2(\alpha)$ | - | | 16.6 | [338/355] | 25.7 | [538/567] | 29.0 |
| | | | | | | | 26.3 |
| $CH_3(\delta)$ | - | | 13.7 | | 13.4 | [5] | 13.57 |
| | | | | | | | 13.55 |

Table 3: $^{13}C$ NMR data in $CDCl_3$ of compounds **1** to **4**; chemical shifts in ppm with respect to TMS; $^{n}J(^{13}C\ ^{117/119}Sn)$ coupling constants in Hz between square brackets.

Mössbauer spectroscopy

The Mössbauer parameters are shown in Table 6. The quadrupole splittings QS are found in the range 3.49-3.90 mm/s. Since Mössbauer spectroscopy is less sensitive to small variations of the tin environment than tin NMR spectroscopy, the two different tin atoms in **4** and **8** could not be discriminated. The Mössbauer parameters are in agreement with a polymeric structure for the triorganotin polyoxaalkanoates, in which tin is five-coordinate with a *trans*-$O_2$ configuration [2]. For the diorganotin polyoxaalkanoates, the QS values conform the structures of figure 1.

Electrospray mass spectroscopy

The monoisotopic mass spectra ($^{1}H$, $^{12}C$, $^{16}O$, $^{120}Sn$) in the cationic mode of water/acetonitrile solutions are reported in Table 7. All compounds are easily complexed by normal or hydrolyzed fragments, or solvent moleculcs. The hydrolyzed species give an indication about stability inside the spectrometer [10]. They are observed for all compounds, but only fragments providing straightforward characterization of M are listed.

| | **5** | | **6** | | **7** | | **8** |
|---|---|---|---|---|---|---|---|
| C(1) | 176.4 | | 175.1 | | 175.8 | | 175.1 |
| CH(i) | 137.8 | | - | | - | | - |
| CH(o) | 136.9 | [47/50] | - | | - | | - |
| CH(p) | 130.2 | [13] | - | | - | | - |
| CH(m) | 128.9 | [62/66] | - | | - | | - |
| $CH_2(8)$ | 72.0 | | 72.0 | | 71.8 | | 72.0 |
| $CH_2(4)(5)$ and (7) | 70.7 | | 70.6 | | 71.1 | | 70.60 |
| | 70.7 | | 70.5 | | 70.6 | | 70.55 |
| | 70.5 | | 70.5 | | 70.4 | | 70.4 |
| $CH_2(2)$ | 69.0 | | 69.0 | | 68.7 | | 69.9 |
| $CH_3(10)$ | 59.0 | | 58.9 | | 59.0 | | 59.0 |
| $CH_2(\beta)$ | - | | 27.8 | [20] | 26.6 | [38] | 27.6 |
| | | | | | | | 27.3 |
| $CH_2(\gamma)$ | - | | 27.0 | [63/66] | 26.3 | [99] | 26.9 |
| | | | | | | | 26.7 |
| $CH_2(\alpha)$ | - | | 16.6 | [339/355] | 25.6 | [540/567] | 29.1 |
| | | | | | | | 25.8 |
| $CH_3(\delta)$ | - | | 13.6 | | 13.5 | | 13.6 |

Table 4: $^{13}C$ NMR data in $CDCl_3$ of compounds **5** to **8**, see legend table 3.

| **1** | **2** | **3** | **4** | **5** | **6** | **7** | **8** |
|---|---|---|---|---|---|---|---|
| -100.0 | 120.7 | -124.7 | -204.8 | -103.2 | 120.7 | -124.1 | -204.9 |
| | | | -215.8 | | | | -217.6 |
| | | | [n.o.] | | | | [119] |

Table 5: $^{117}Sn$ NMR data in $CDCl_3$ of compounds **1** to **8**; chemical shifts in ppm with respect to $(CH_3)_4Sn$; coupling constants $^2J(^{117}Sn-^{117/119}Sn)$ in Hz in square brackets; n.o.: not observed.

| | QS | IS | $\Gamma_1$ | $\Gamma_2$ |
|---|---|---|---|---|
| **1** | 3.60 | 1.24 | 0.85 | 0.79 |
| **2** | 3.81 | 1.47 | 1.15 | 1.14 |
| **3** | 3.90 | 1.44 | 1.28 | 1.02 |
| **4** | 3.42 | 1.34 | 1.22 | 1.18 |
| **5** | 3.44 | 1.29 | 0.91 | 0.87 |
| **6** | 3.84 | 1.47 | 1.07 | 1.02 |
| **7** | 3.77 | 1.42 | 1.36 | 1.18 |
| **8** | 3.49 | 1.32 | 0.90 | 0.90 |

Table 6: $^{119m}Sn$ Mössbauer parameters: QS (mm/s) quadrupole splitting, IS (mm/s) isomer shift relative to $Ca^{119}SnO_3$, $\Gamma_1$ and $\Gamma_2$ (mm/s) line width.

Elemental analysis

C and H elemental analysis was achieved for all compounds (see table 8).

Compounds **2** and **6** contain water in a (2/1) tributyltin carboxylate/water ratio. The dimeric compounds **4** and **8** are found to contain two molecules of water per dimeric distannoxane unit. The presence of water is confirmed by the proton NMR spectra (see Tables 1 and 2).

*In vitro antitumour screening*

All compounds were screened against seven tumoural cell lines of human origin.

The $ID_{50}$ values are reported in Table 9 and compared to those of some drugs with clinical applications and of organotin carboxylates containing crown ether moieties: di-n-butyl, tri-n-butyl and triphenyltin derivatives of 4-carboxybenzo-18-crown-6 and -15-crown-5 [11] of which the structures are depicted in figure 2. The two series (**1** to **4** and **5** to **8**) show pairwise comparable activities.

| fragment | 1 | 5 |
|---|---|---|
| M + $H^+$ | 485; 2% | 529; 6% |
| M + $Ph_3Sn^+$ | 835; 100% | 879; 100% |
| | **2** | **6** |
| M + $H^+$ | 425; 2% | |
| M + $Bu_3Sn^+$ | 715; 100% | 759; 100% |
| | **3** | **7** |
| $[RCOOSnBu_2]^+$ | 367; 9% | 411; 13% |
| M + $H^+$ | 501; 5% | 589; 23% |
| | **4** | **8** |
| $[RCOOBu_2SnOSnBu_2]^+$ | 617; 100% | 661; 100% |
| $[RCOOBu_2SnOSnBu_2OSnBu_2]^+$ | 867; 62% | 911; 70% |
| $RCOOBu_2SnOSnBu_2OCOR + [Bu_2SnOH]^+$ | 1001; 40% | 1089; 33% |

Table 7: Characteristic electrospray fragment-ions for compounds **1** to **8**.

| | structure | C | H |
|---|---|---|---|
| **1** | $C_{23}H_{24}SnO_4$ | 57.2 [57.39] | 5.0 [4.67] |
| **2** | $C_{17}H_{36}SnO_4{\cdot}1/2H_2O$ | 47.3 [47.35] | 8.6 [8.57] |
| **3** | $C_{18}H_{36}SnO_8$ | 43.3 [43.40] | 7.3 [7.48] |
| **4** | $C_{52}H_{108}Sn_4O_{18}{\cdot}2H_2O$ | 40.8 [40.72] | 7.4 [7.21] |
| **5** | $C_{25}H_{28}SnO_5$ | 57.0 [57.06] | 5.4 [5.36] |
| **6** | $C_{19}H_{40}SnO_5{\cdot}1/2H_2O$ | 47.9 [47.70] | 8.7 [8.78] |
| **7** | $C_{22}H_{44}SnO_{10}$ | 45.0 [44.80] | 7.6 [7.76] |
| **8** | $C_{60}H_{124}Sn_4O_{22}{\cdot}2H_2O$ | 42.2 [42.15] | 7.6 [7.44] |

Table 8: Elemental analysis of compounds **1** to **8** (found [calculated]).

| | MCF-7 | EVSA-T | WiDr | IGROV | M19MEL | A 498 | H 226 |
|---|---|---|---|---|---|---|---|
| **1** | 13 | 12 | 34 | 37 | 31 | 32 | 33 |
| **2** | 32 | 40 | 82 | 84 | 90 | 153 | 61 |
| **3** | 60 | 62 | 379 | 128 | 115 | 134 | 161 |
| **4** | <3 | <3 | 6 | <3 | <3 | <3 | 5 |
| **5** | 9 | 9 | 19 | 33 | 24 | 21 | 25 |
| **6** | 36 | 25 | 40 | 89 | 78 | 93 | 56 |
| **7** | 86 | 66 | 495 | 178 | 167 | 145 | 280 |
| **8** | <3 | <3 | 3 | <3 | <3 | <3 | <3 |
| **9** | 15 | 12 | 13 | 30 | 16 | 43 | 37 |
| **10** | 35 | 6 | 11 | 30 | 70 | 97 | 100 |
| **11** | 155 | 128 | 781 | 260 | 219 | 282 | 281 |
| **12** | 36 | 46 | 239 | 82 | 68 | 126 | 73 |
| **13** | <3 | <3 | <3 | <3 | <3 | <3 | <3 |
| **14** | <3 | <3 | <3 | <3 | <3 | <3 | <3 |
| **15** | 273 | 237 | 332 | 321 | 286 | 49 | 854 |
| cisplatin | 699 | 422 | 967 | 169 | 558 | 2253 | 3269 |
| doxorubicine | 10 | 8 | 11 | 60 | 16 | 90 | 199 |
| etoposide | 2594 | 317 | 150 | 580 | 505 | 1314 | 3934 |
| 5-fluorouracil | 750 | 475 | 225 | 297 | 442 | 143 | 340 |
| methotrexate | 18 | 5 | <3 | 7 | 23 | 37 | 2287 |

Table 9: *In vitro* inhibition doses $ID_{50}$ (in ng/ml) against seven cancer cell lines of human origin, MCF-7 and EVSA-T, two breast cancers; WiDr, a colon carcinoma; IGROV, an ovarian cancer; M19 MEL, a melanoma, A 498 a renal cancer and H 226, a non small cell lung cancer. The structures of compounds **9** to **15** are depicted in figure 2.

Compounds **4** and **8** exhibit exceptionally low inhibition doses and are comparable to those of compounds **13** and **14**. Activities of **3** and **7** are very different from those of **4** and **8**. The former can easily be hydrolyzed into the latter as was already announced previously. Hydrolysis of **4** and **8** has no determinant effect on *in vitro* antitumoural activity.

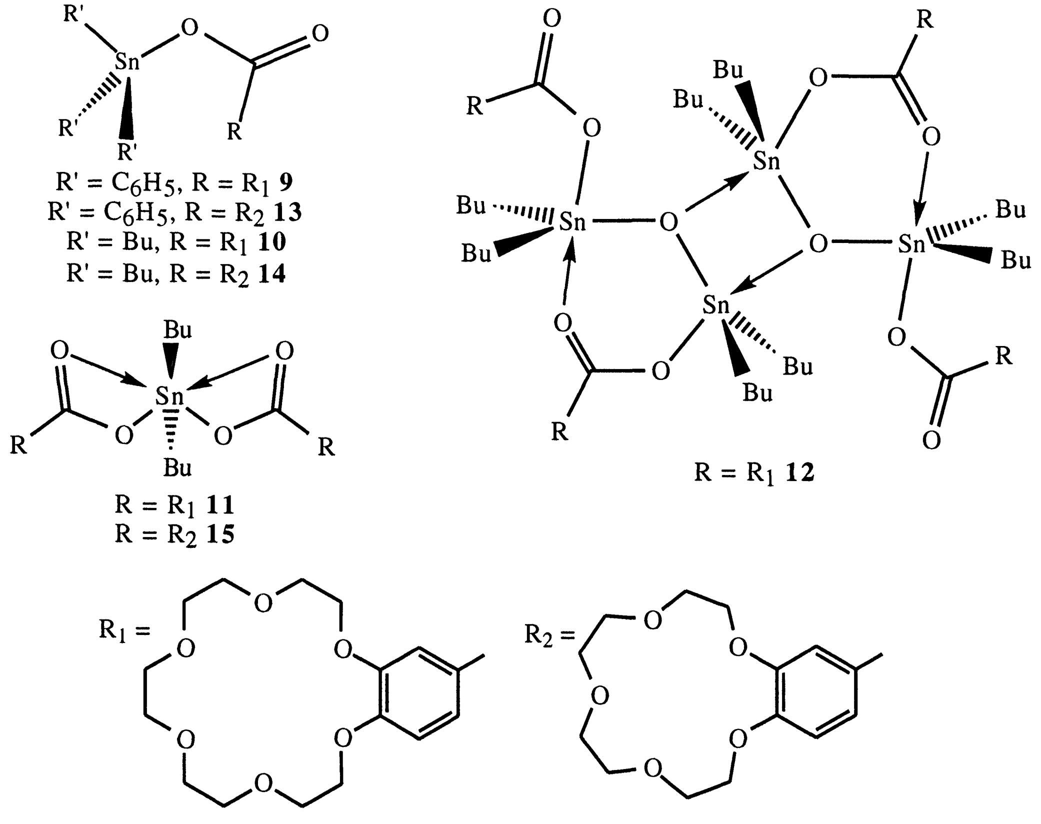

*figure 2*

**Experimental part**

*Synthesis*

Triorganotin carboxylates

Compounds **1** and **5** are typically prepared by mixing equimolar quantities of triphenyltin hydroxide and the desired polyoxacarboxylic acid in 250 ml benzene in a 500 ml flask equipped with a Dean-Stark funnel. The mixture is refluxed for 8 to 12 hours. The binary azeotrope benzene/water is distilled off up to 50% to the initial solvent volume. The remaining solution is evaporated under vacuum.
The synthesis with tri-n-butyltin acetate yielding **2** and **6** is analog.

| | m.p. | yield | purification method |
|---|---|---|---|
| **1** | 100-102°C | 95% | recystallization from hexane/chloroform |
| **2** | liquid | 95% | Sephadex LH-20; elution with methylene chloride |
| **3** | liquid | 98% | no purification |
| **4** | liquid | 80% | Sephadex LH-20; elution with methylene chloride |
| **5** | 109-111°C | 92% | recrystallization from diethylether/methylene chloride |
| **6** | liquid | 92% | Sephadex LH-20; elution with methylene chloride |
| **7** | liquid | 95% | no purification |
| **8** | liquid | 80% | Sephadex LH-20; elution with methylene chloride |

Table 10: Synthesis data for compounds **1** to **8**.

Diorganotin carboxylates

Di-n-butyltin oxide is allowed to react with 1-propanol (typically 1g of di-butyltin oxide and 4 ml of 1-propanol) in benzene (250 ml) in a 500 ml three-necked flask. The reaction mixture is refluxed for 3 hours under elimination of the ternary azeotrope benzene/water/1-propanol (Dean-Stark). After cooling to room temperature, the appropriate amount of polyoxaalkanecarboxylic acid (1/1 molar ratio polyoxaalkanecarboxylic acid / di-n-butyltin oxide for **3** and **7**; 2/1molar ratio for **4** and **8**) is added slowly and the mixture is stirred overnight. The solvent is then evaporated and the product treated as indicated in Table 10 in which all experimental synthesis details are summarized.

*NMR measurements*

All 2D NMR spectra and some of the 1D spectra were recorded on a Bruker AMX500 spectrometer interfaced with a X32 computer and operating at 500.13 and 125.77 MHz for the $^{1}H$ and $^{13}C$ nuclei, respectively. All NMR spectra except $^{1}H$ and $^{13}C$ of compound **4** were acquired on a Bruker AC250 instrument equipped with a Quattro probe tuned to 250.13, 62.93 and 89.15 MHz for $^{1}H$, $^{13}C$ and $^{117}Sn$ nuclei respectively. $^{1}H$ and $^{13}C$ chemical shifts were referenced to the standard $Me_4Si$ scale from respectively residual $^{1}H$ and $^{13}C$-$^{2}H$ solvent resonances of chloroform ($CHCl_3$, 7.23 and $CDCl_3$, 77.0 ppm for $^{1}H$ and $^{13}C$ nuclei, respectively). The $^{117}Sn$ resonance frequencies were set from the absolute reference $\Xi(^{117}Sn)$ = 35.632295 MHz[12,13]. 2D gradient enhanced $^{1}H$-$^{13}C$ HMQC[14] and HMBC[15] correlation spectra were acquired using the pulse sequences of the Bruker program library, adapted to include gradient pulses,[16-19] as described recently[20].

*Mössbauer spectroscopy*

The Mössbauer spectra were recorded as described elsewhere[21].

*Mass spectrometry*

The electrospray mass spectra were recorded in the cationic mode on a Micromass Quattro II instrument coupled with a Masslynx system (ionisation in an electric field of 3,5 kV; source temperature: 80°C; source pressure: 1 atm; analyzer pressure: $10^{-5}$ mbar)[22,23]. Cone voltages were 30 V for compounds **1** to **3** and **5** to **7**, and 70 V for compounds **4** and **8**.

*Antitumour screening*

The protocol followed for the antitumour screenings has already been reported[24,25].

**Acknowledgements**

We thank Mrs. I. Verbruggen and Mr. L. Ghys for recording NMR spectra, Dr. G. Laus and Mr. M. Desmet for the mass spectra and Prof. Dr. B. Mahieu for the Mössbauer spectra. We are grateful to Mr. H. J. Kolker, Dr. J. Verweij, Prof. Dr. G. Stoter, Dr. J. H. M. Schellens, Laboratory of Experimental Chemotherapy and Pharmacology, Department of Medical Oncology, Rotterdam Cancer Institute, NL - 3008 AE, Rotterdam, The Netherlands, for performing the *in vitro* screenings. This research was supported by the "Ministère de l'Education Nationale du Luxembourg" (grants nr BFR93/051, BPU96/130 and BPU97/138, M. K.), the "Action Lions Vaincre le cancer du Luxembourg" (M. K), the Belgian "Nationaal Fonds voor Wetenschappelijk Onderzoek" (N.F.W.O. grant nr G.0054.96, M. G.), and the Fund for Scientific Research Flanders (Belgium, grant nr G.0192.98, R. W., M. B.).

**References**

[1] M. Gielen, Tin-based antitumour drugs, *Coord. Chem. Rev.* **151** (1996), 41-51.
[2] R. Willem, A. Bouhdid, M. Biesemans, J. C. Martins, D. de Vos, E. R. T. Tiekink, M. Gielen, *J. Organomet. Chem.* **541** (1996), 203; R. Willem, A. Bouhdid, B. Mahieu, L. Ghys, M. Biesemans, E. R. T. Tiekink, D. de Vos, M. Gielen, *J. Organomet. Chem.* **531** (1997), 151.
[3] G. Atassi, *Rev. Si, Ge, Sn, Pb cpds.* **8** (1985), 219.
[4] D. J. Cram, G. S. Hammond, in "Organic Chemistry", McGraw-Hill Book Company, NY, (1959), pp. 158-162.
[5] M. Gielen, A. El Khloufi, M. Biesemans, R. Willem, *Appl. Organomet. Chem.* **7** (1993), 119.
[6] A. G. Davies, D. C. Kleinschmidt, P. R. Palan, S. C. Vasishtha, *J. Chem. Soc. (C)* (1971), 3972.
[7] D. L. Alleston, A. G. Davies, M. Hancock, R. F. M. White, *J. Chem. Soc.* (1963), 5469.
[8] B. Wrackmeyer, *Annu. Rep. NMR Spectrosc.* **16** (1985), 73.
[9] B. Wrackmeyer, in "Advanced Applications of NMR to Organometallic Chemistry", Ed.: M. Gielen, R. Willem, B. Wrackmeyer, J. Wiley, Chichester (1996), pp. 87-122.
[10] W. Henderson, M. J. M. Taylor, *Polyhedron* **15** (1996), 1957.
[11] M. Kemmer, M. Gielen, M. Biesemans, D. de Vos, R. Willem, *unpublished results.*
[12] J. Mason, Multinuclear NMR, Plenum Press, NY, (1987), pp. 625-629.

[13] A. G. Davies, P. G. Harrison, J. D. Kennedy, R. J. Puddephatt, T. N. Mitchell, W. J. McFarlane, *J. Chem. Soc. (A)* (1969), 1136.
[14] A. Bax, R. H. Griffey, B. H. Hawkins, *J. Magn. Reson.* **55** (1983), 301.
[15] A. Bax, M. F. Summers, *J. Magn. Reson.* **67** (1986), 565.
[16] J. Keeler, R. T. Clowes, A. L. Davis, E. D. Laue, *Meth. Enzymol.* **239** (1994), 145.
[17] J.-M. Tyburn, I. M. Bereron, D. M. Doddrell, *J. Magn. Reson.* **97** (1992), 305.
[18] J. Ruiz-Cabello, G. W. Vuister, C. T. W. Moonen, P. Van Gelderen, J. S. Cohen, P. C. M. Van Zijl, *J. Magn. Reson.* **100** (1992), 282.
[19] G. W. Vuister, R. Boelens, R. Kaptein, R. E. Hurd, B. K. John, P. C. M. Van Zijl, *J. Am. Chem. Soc.* **113** (1991), 9688.
[20] R. Willem, A. Bouhdid, F. Kayser, A. Delmotte, M. Gielen, J. C. Martins, M. Biesemans, B. Mahieu, E. R. T. Tiekink, *Organometallics* **15** (1996), 1920.
[21] M. Bouâlam, R. Willem, M. Biesemans, B. Mahieu, J. Meunier-Piret, M. Gielen, *Main Group Met. Chem.* **14** (1991), 41.
[22] G. Lawson, R. H. Dahm, N. Ostah, E. D. Woodland, *Appl. Organomet. Chem.* **10** (1996), 125.
[23] G. Lawson, N. Ostah, *Appl. Organomet. Chem.* **8** (1994), 525.
[24] Cancer: principles and practice of oncology, Eds.: V. T. DeVita Jr., S. Hellman, S. A. Rosenberg, 5th Ed. (1997), Lippincott-Raven Publ., Philadelphia.
[25] Y. P. Kepers, G. J. Peters, J. Van Ark-Otte, B. Winograd, H. M. Pinedo, *Eur. J. Cancer* **27** (1991), 897.

**Received: June 23, 1998 - Accepted: July 14, 1998**

